# Reproducibility of magnetic resonance fingerprinting-based T_1_ mapping of the healthy prostate at 1.5 and 3.0 T: A proof-of-concept study

**DOI:** 10.1371/journal.pone.0245970

**Published:** 2021-01-29

**Authors:** Nikita Sushentsev, Joshua D. Kaggie, Rhys A. Slough, Bruno Carmo, Tristan Barrett

**Affiliations:** 1 Department of Radiology, Addenbrooke’s Hospital and University of Cambridge, Cambridge, United Kingdom; 2 CamPARI Prostate Cancer Group, Addenbrooke’s Hospital and University of Cambridge, Cambridge, United Kingdom; Fondazione Istituto G.Giglio di Cefalu, ITALY

## Abstract

Facilitating clinical translation of quantitative imaging techniques has been suggested as means of improving interobserver agreement and diagnostic accuracy of multiparametric magnetic resonance imaging (mpMRI) of the prostate. One such technique, magnetic resonance fingerprinting (MRF), has significant competitive advantages over conventional mapping techniques in terms of its multi-site reproducibility, short scanning time and inherent robustness to motion. It has also been shown to improve the detection of clinically significant prostate cancer when added to standard mpMRI sequences, however, the existing studies have all been conducted on 3.0 T MRI systems, limiting the technique’s use on 1.5 T MRI scanners that are still more widely used for prostate imaging across the globe. The aim of this proof-of-concept study was, therefore, to evaluate the cross-system reproducibility of prostate MRF T_1_ in healthy volunteers (HVs) using 1.5 and 3.0 T MRI systems. The initial validation of MRF T_1_ against gold standard inversion recovery fast spin echo (IR-FSE) T_1_ in the ISMRM/NIST MRI system revealed a strong linear correlation between phantom-derived MRF and IR-FSE T_1_ values was observed at both field strengths (R^2^ = 0.998 at 1.5T and R^2^ = 0.993 at 3T; p = < 0.0001 for both). In young HVs, inter-scanner CVs demonstrated marginal differences across all tissues with the highest difference of 3% observed in fat (2% at 1.5T vs 5% at 3T). At both field strengths, MRF T_1_ could confidently differentiate prostate peripheral zone from transition zone, which highlights the high quantitative potential of the technique given the known difficulty of tissue differentiation in this age group. The high cross-system reproducibility of MRF T_1_ relaxometry of the healthy prostate observed in this preliminary study, therefore, supports the technique’s prospective clinical validation as part of larger trials employing 1.5 T MRI systems, which are still widely used clinically for routine mpMRI of the prostate.

## Introduction

Multiparametric magnetic resonance imaging (mpMRI) has recently been adopted by major European and American prostate cancer (PCa) guidelines as the key diagnostic tool for detection, local staging and active surveillance of the disease [1, 2]. Despite significant advantages over the traditional systematic biopsy pathway, such as high negative predictive value for clinically significant PCa, mpMRI has limitations including a known learning curve and considerable interobserver variation for image assessment, even amongst experience readers [3–8]. Making prostate MRI more objective by facilitating clinical translation of quantitative imaging techniques is viewed by many experts as key to solving the aforementioned challenges [9, 10]. This move is also supported by the Prostate Imaging Reporting and Data System (PI-RADS) steering committee with a view to complement the current recommendations that are based exclusively on qualitative visual assessment of images [11, 12].

MR fingerprinting is a rapidly developing quantitative imaging technique enabling fast and simultaneous generation of tissue T_1_ and T_2_ property maps that are inherently spatially registered and robust to motion [13, 14]. The key competitive advantages of MRF over standard mapping techniques, which also make it attractive for PCa imaging, include its higher multi-site reproducibility and significantly shorter acquisition time, which is important given the current move towards developing abbreviated prostate imaging protocols in order to reduce scanning times whilst maintaining quality [14–19].

Several pilot studies investigating the added value of MRF to prostate mpMRI have demonstrated significantly improved detection of clinically significant peripheral zone (PZ) and transition zone (TZ) tumors when MRF was used in combination with apparent diffusion coefficient (ADC) mapping [20–23]. However, wider clinical validation of MRF as part of an mpMRI protocol is limited by the fact studies have only validated performance on 3T MRI systems, whist the majority of prostate mpMRI scans even in developed countries are still performed on 1.5T scanners [24–27]. The most recent version of the PI-RADS guidelines (v2.1) suggest that both 1.5T and 3T allow for reliable diagnostic exams in the context of adequate hardware and software, and when optimal acquisition parameters are ensured. [10]

Whilst MRF at both 1.5T and 3T has been used in cardiac and brain imaging, there are surprisingly few studies dedicated to cross-system validation of MRF in different body areas other than the brain [28–30]. It is known that longitudinal T_1_ relaxation times are more sensitive to an increase of the magnetic field strength compared to T_2_ relaxation and quantitative magnetisation transfer parameters [31]. In the prostate, which generally has longer T_1_ values compared to other pelvic and abdominal organs, field-dependent variation of T_1_ can reach up to 24%, which may affect the cross-system reproducibility of MRF [15, 32].

Therefore, the objective of this proof-of-concept study was to evaluate the cross-system reproducibility of MRF-based T_1_ mapping of the healthy prostate with data acquired from both phantoms and healthy volunteers using 1.5T and 3T MRI systems.

## Methods

### Phantom study

To evaluate the accuracy of the introduced T_1_ and T_2_ measurements, MRF and standard relaxation mapping data were obtained from the ISMRM/NIST phantom. [27] Phantom data were obtained on 1.5T MR450 and 3T MR750w scanners (both GE Healthcare, Waukesha, WI, USA) using a 32-channel receiver coil. Regions-of-interest (ROIs) were created from the spheres in either the T_1_ or T_2_ layer of the phantom. MR Fingerprinting was performed as further described in section 2.4.

Conventional T_1_ and T_2_ mapping was performed with inversion recovery fast spin echo (IR-FSE) and multiple spin echo (MSE) imaging, respectively. The field-of-view (FOV) = 260x260 mm^2^, matrix = 256x256 and slice thickness = 3 mm matched in all techniques. For 2D IR-FSE T_1_ mapping, data were acquired with inversion times (TIs) of 50, 100, 200, 400, 800, 1600, and 2400 ms. The IR-FSE sequence used a repetition time (TR) = 8000 ms and echo time (TE) = 13 ms at both 1.5 T and 3.0 T. T_1_ data for all non-MRF techniques were fit using non-linear fitting of the signal equations in Python. Multiple spin echo (MSE) data for T_2_ estimations were acquired with TEs = 8.1, 16.3, 24.4, 32.6, 40.7, 58.9, 57.0, 65.2 ms. MSE data were fit with a log linear least squares algorithm. The acquisition matched the FOV, matrix, and slices as the MRF acquisition.

### Healthy volunteers study

All elements of this prospective study were carried out in accordance with the Declaration of Helsinki and were approved by the institutional ethics board (NRES Committee East of England, UK), with written informed consent obtained from all participants. The volunteers were aged at least 18 years were included in this study, with the exclusion criteria being previous diagnosis or treatment for prostate cancer and clinical contraindication to MRI.

### Anatomical imaging

Healthy volunteers underwent prostate MRI on the same 1.5T MR450 and 3T MR750 scanners (both GE Healthcare, Waukesha, WI, USA) used in the phantom study. As per clinical mpMRI acquisition, the protocol included Axial T1 and multiplanar high-resolution T2-weighted 2D fast recovery FSE (FOV = 18x18 cm; voxel size 0.35x0.35 mm2; slice thickness = 3–4 mm; gap = 0–0.5 mm). Diffusion-weighted imaging (DWI) was performed using a spin-echo echo-planar imaging pulse sequence (FOV 28cm; slice thickness 3mm; gap 0mm; b-values: b-150, b-750, and b-1,400 s/mm^2^) and an additional small FOV (24 cm) b-2,000s/mm^2^ DWI sequence; apparent diffusion coefficient (ADC) maps were calculated automatically.

### MR fingerprinting protocol

MRF at both 1.5 T and 3.0 T was acquired with an inversion-prepared 2D steady-state-free-precession (SSFP) MRF sequence [13]. The acquisition consisted of 979 undersampled interleaved spirals with 656 points per spiral, and with sequential spirals rotated by the golden angle. The maximum gradient strength per spiral was 28 mT/m and the maximum slew rate was 108 T/m/s. The imaging parameters were: field-of-view (FOV) = 260x260 mm^2^, matrix = 256x256, slices = 3 for ISMRM/NIST imaging and 12–14 for *in vivo* imaging, *in vivo* slice thickness = 6 mm and 3 mm at 1.5T and 3T, respectively, spacing = 5mm and 6mm at 1.5T and 3T (39 mm for both for ISMRM/NIST imaging), respectively, sampling bandwidth = ±250 kHz, slice dephasing = 8π, echo time (TE) = 2.5 ms, repetition time (TR) = 10 ms, acquisition time = 9.79 seconds/slice. The flip angle lists matched those in Jiang et al. [33]. Axial images were acquired to match the standard acquisition planes for other prostate image assessments.

### Image analysis

MRF T_1_ values for the whole prostate, peripheral zone (PZ), transition zone (TZ), internal obturator muscle and adipose tissue in the ischioanal fossa were calculated from ROIs drawn by a single fellowship-trained uro-radiologist using the open-source segmentation software ITK-SNAP [34]. ROIs were drawn using anatomical T2WI as reference and subsequently transposed on to the MRF T_1_ with their size and location being matched to the appropriate FOV parameters and anatomical position of the outlined structures using in-house software developed within Python using the PyQtGraph and PyDicom libraries [35].

### Statistics

In the phantom study, simple linear regression was used to evaluate the relationship between T_1_ and T_2_ values obtained using IR-FSE, MSE and MRF mapping techniques with their agreement assessed using Bland-Altman analysis. In the healthy volunteers study, the D’Agostino and Pearson test was applied to assess the distribution of imaging values with their intergroup comparison performed using either paired t-test or Wilcoxon matched-pairs signed rank test as appropriate. Both in the phantom and healthy volunteers studies, the variation of imaging values as a means of their cross-system reproducibility was evaluated using a coefficient of variation (CV). All plots and figures were created in Prism 8 (GraphPad Software, San Diego, CA).

## Results

### Phantom study

Summary mean inter-scanner T_1_ and T_2_ values obtained from the corresponding ISMRM/NIST phantom layers using MRF, inversion recovery fast spin echo (IR-FSE) and multiple spin echo (MSE) imaging are presented in **Table 1**. Paired t-test showed no inter-scanner differences between IR-FSE and MRF-based T_1_ phantom values (p = 0.795 and p = 0.102, respectively). As a measure of reproducibility, coefficients of variations (CVs) were calculated and are also presented in **Table 1**, showing inter-scanner differences of 0.17% and 2.57% for IR-FSE and MRF T_1_, respectively. Inter-scanner T_2_ values obtained using MRF and MSE imaging were, however, significantly different in both cases (p = 0.0002 and p = 0.0006, respectively) with differences in their CVs reaching 0.10% and 7.66%, respectively.

**Table 1:** Summary ISMRM/NIST phantom T_1_ and T_2_ relaxation times and coefficients of variation obtained using inversion recovery fast spin echo (IR-FSE), multiple spin echo (MSE) and magnetic resonance fingerprinting (MRF) at 1.5T and 3T systems.

	Mean ± SD			Coefficient of variation (%)	
Sequence	1.5T	3T	*P*	1.5T	3T
**IR-FSE T_1_**	616.1 ± 400.9	615.8 ± 401.8	0.7951	65.08	65.25
**MRF T_1_**	559.3 ± 402.2	544.1 ± 405.3	0.1019	71.91	74.48
**MSE T_2_**	137.8 ± 112.2	92.8 ± 82.7	0.0002	116.80	116.90
**MRF T_2_**	471.2 ± 550.2	95.6 ± 111.8	0.0006	81.46	89.12

SD = standard deviation.

**Fig 1:**
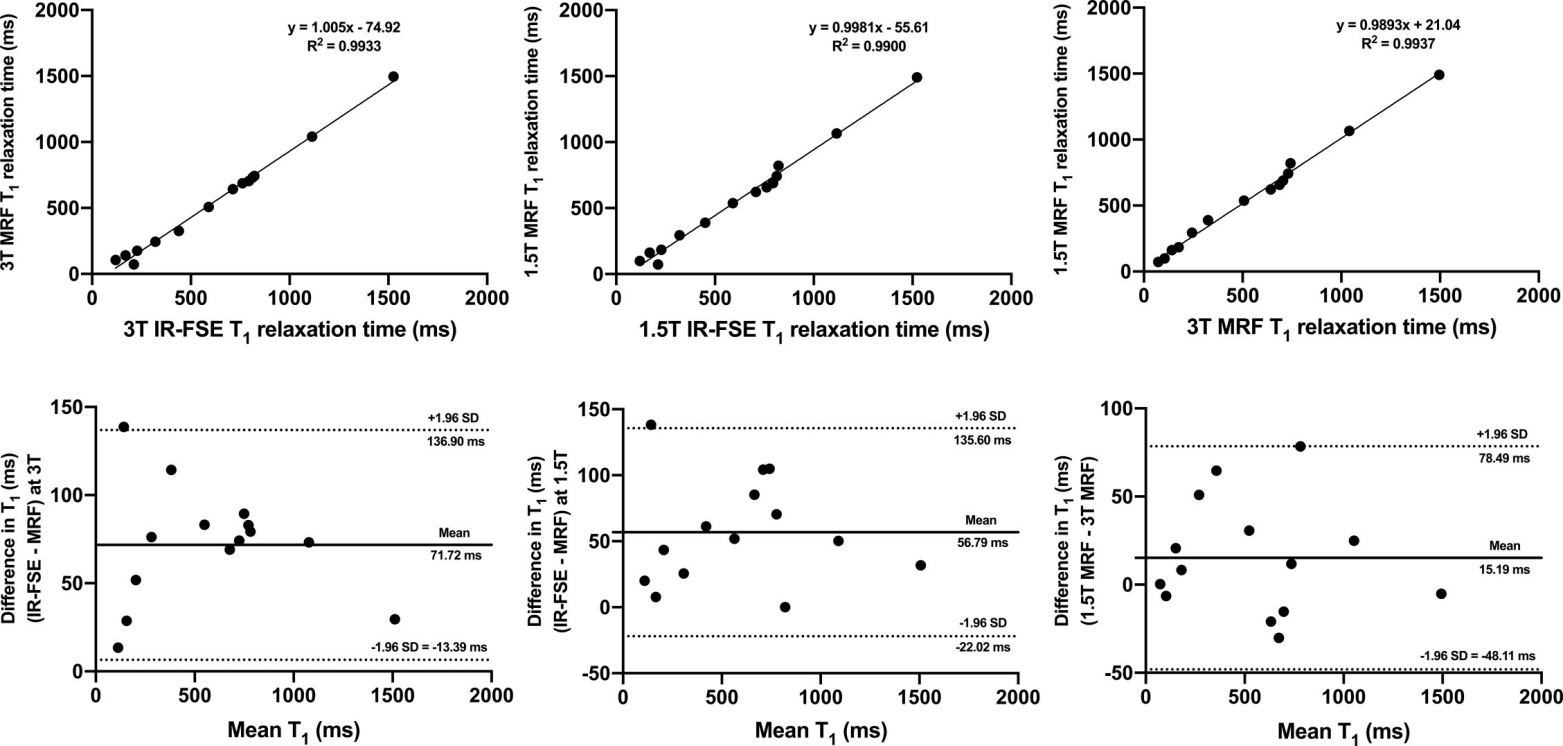
Linear regression plots (a-c) and Bland-Altman plots (d-f) comparing MRF T_1_ ISMRM/NIST phantom values with those obtained using IR-FSE at 3T (a, d) and 1.5T (b, e). An inter-scanner comparison of MRF T_1_ values is also presented (c, f). On Fig (d-f), dotted lines represent upper and lower 95% limits of agreement and bold lines represent the mean biases with appropriate captions included. MRF = magnetic resonance fingerprinting, IR-FSE = inversion recovery fast spin echo, SD = standard deviation.

**Fig 1A** and **1B** show the mean ISMRM/NIST phantom MRF T_1_ values plotted against T_1_ values obtained using inversion recovery fast spin echo (IR-FSE) imaging at 1.5T and 3T, respectively. The analysis showed a strong linear correlation between MRF and IR-FSE at both field strengths (R^2^ = 0.998 at 1.5T and R^2^ = 0.993 at 3T; p = < 0.0001 for both) with slopes of the linear fits and y-intercepts presented in the corresponding figures. The Bland-Altman analysis revealed good agreement between the two techniques at both 1.5T and 3T with the corresponding mean bias and 95% limits of agreement (LOA) presented in **Fig 1D** and **1E**, respectively. When plotted against each other, MRF T_1_ values obtained at 1.5T and 3T also demonstrated a strong linear correlation (R^2^ = 0.994; p < 0.0001) and acceptable agreement (bias 15.19 ms, 95% LOA -48.11 ms to 78.49 ms) with corresponding plots presented in **Fig 1C** and **1F**, respectively.

**Fig 2:**
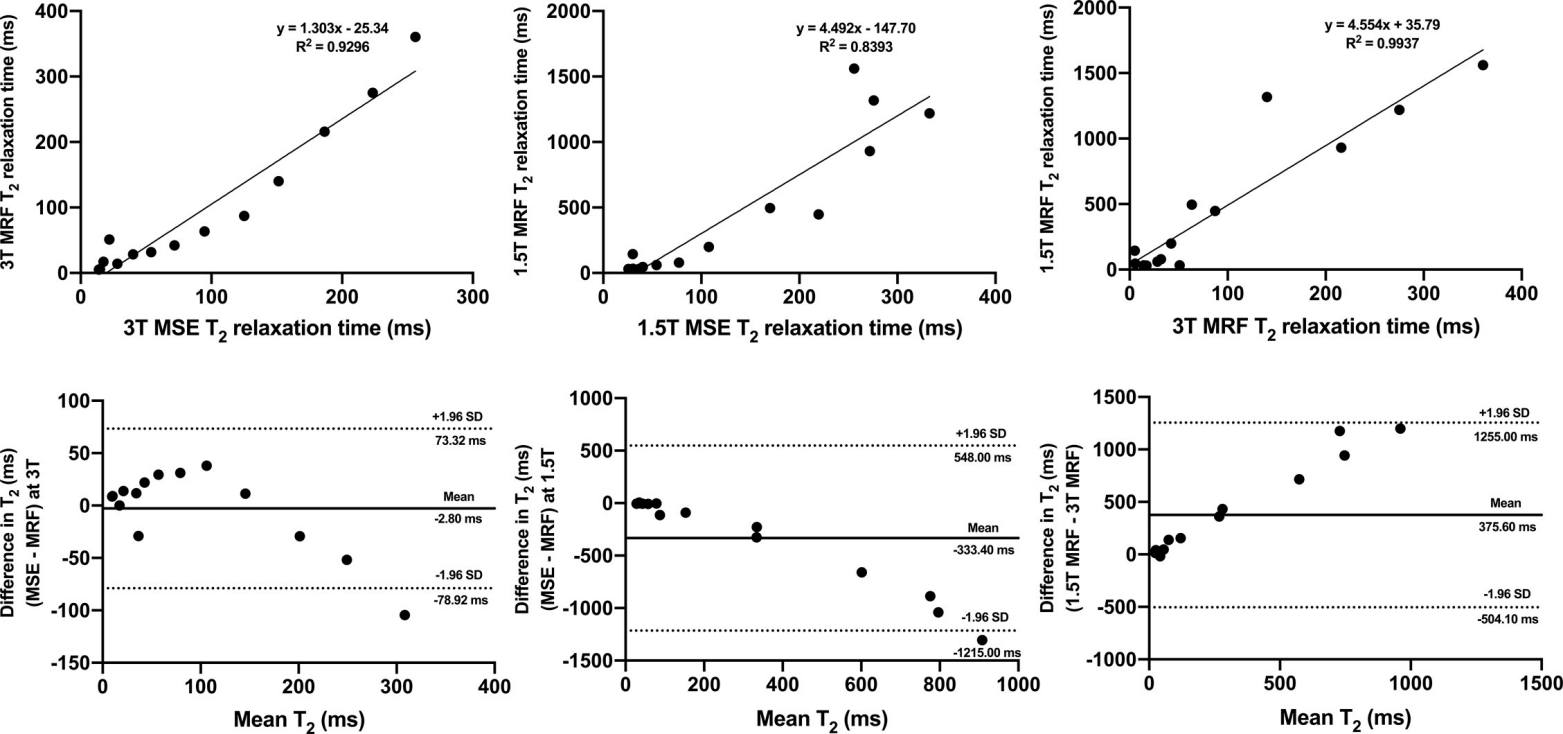
Linear regression plots (a-c) and Bland-Altman plots (d-f) comparing MRF T_2_ ISMRM/NIST phantom values with those obtained using MSE at 3T (a, d) and 1.5T (b, e). An inter-scanner comparison of MRF T_2_ values is also presented (c, f). On Fig (d-f), dotted lines represent upper and lower 95% limits of agreement and bold lines represent the mean biases with appropriate captions included. MRF = magnetic resonance fingerprinting, MSE = multiple spin echo, SD = standard deviation.

The outcomes of a similar analysis comparing the MRF and multiple spin echo (MSE) T_2_ values obtained at 1.5T and 3T are presented in **Fig 2**. It is of note that T_2_ values obtained in this study showed consistently poorer reproducibility as demonstrated by both linear regression and Bland-Altman analysis, prompting us to focus the following presentation and analysis of the *in vivo* results on T_1_ relaxometry.

### Healthy volunteers study

The study included 10 healthy male volunteers (mean age 32.3 years, range 24–41) who underwent MRF of the prostate at 1.5T and 3T within the same imaging session.

Summary mean inter-scanner MRF T_1_ values and coefficients of variation (CVs) obtained from the whole prostate, peripheral zone (PZ), transition zone (TZ), internal obturator muscle and fat in the ischioanal fossa are presented in **Table 2**. As expected, 3T MRF T_1_ values were significantly longer in all tissues compared to 1.5T MRF (*P* < 0.0001 for all). Inter-scanner CVs demonstrated only marginal differences in all tissues with the highest difference of 3% observed in fat (2% at 1.5T vs 5% at 3T). T_1_ values obtained from the PZ showed the highest overall variation with CVs at 1.5T and 3T being 10.9% and 10.3%, respectively. The outcomes of a similar analysis conducted for *in vivo* MRF T_2_ relaxometry is presented in **S1 Table**.

**Table 2:** Summary MRF T_1_ relaxation times and coefficients of variation obtained at 1.5T and 3T from the whole prostate, peripheral zone, transition zone, internal obturator muscle and fat from the ischioanal fossa.

	Mean ± SD			Coefficient of variation (%)	
Tissue	1.5T	3T	*P*	1.5T	3T
**Prostate**	1054.0 ± 80.1	1769.0 ± 112.7	< 0.0001	7.60	6.37
**Peripheral zone**	1202.0 ± 131.3	2041.0 ± 209.4	< 0.0001	10.93	10.26
**Transition zone**	968.8 ± 60.7	1552.0 ± 105.2	< 0.0001	6.26	6.78
**Muscle**	725.6 ± 23.8	1446.0 ± 73.95	< 0.0001	3.29	5.12
**Fat**	192.1 ± 3.8	345.1 ± 17.1	< 0.0001	1.99	4.96

SD = standard deviation.

**Fig 3:**
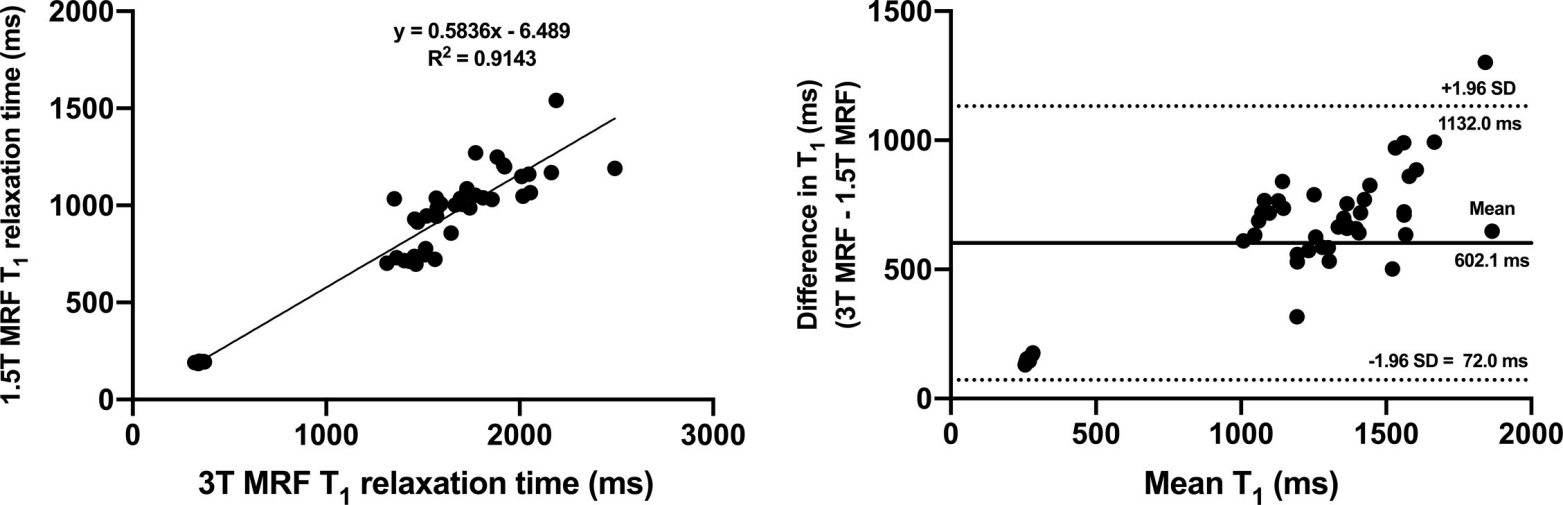
Linear regression (a) and Bland-Altman (b) plots comparing in vivo MRF T_1_ values obtained from all tissues combined at 1.5T and 3T systems. On Fig (b), dotted lines represent upper and lower 95% limits of agreement and bold lines represent the mean biases with appropriate captions included. MRF = magnetic resonance fingerprinting, SD = standard deviation.

**Fig 4:**
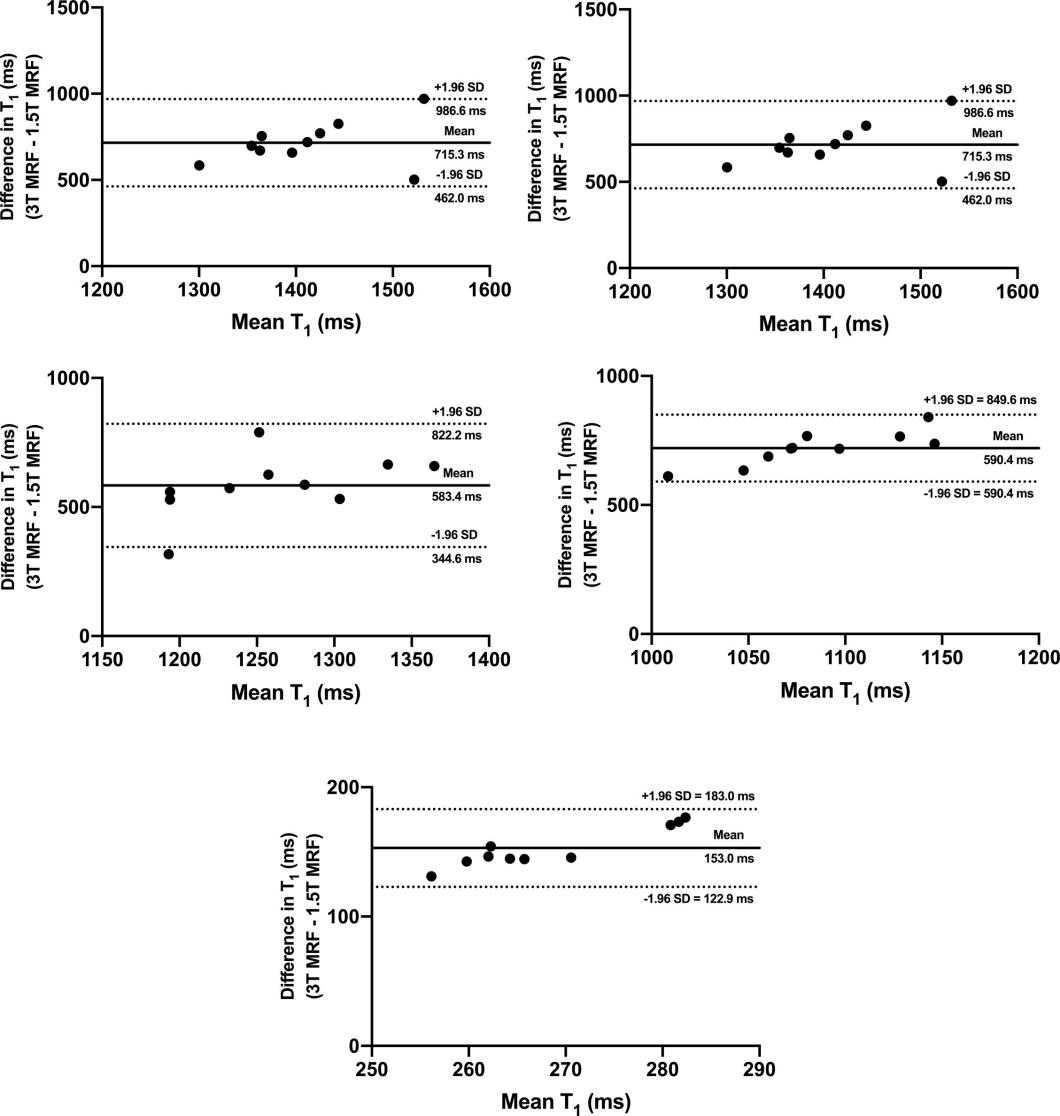
Bland-Altman plots comparing in vivo MRF T_1_ values obtained from the whole prostate (a), peripheral zone (b), transition zone (c), internal obturator muscle (d) and fat in the ischioanal fossa (e) at 1.5T and 3T systems. Dotted lines represent upper and lower 95% limits of agreement and bold lines represent the mean biases with appropriate captions included. MRF = magnetic resonance fingerprinting, SD = standard deviation.

Albeit weaker than in the phantom experiment, a strong linear correlation was noted between the combined *in vivo* 1.5T and 3T MRF T_1_ values obtained from all tissues included in the analysis (R^2^ = 0.914; p < 0.0001); **Fig 3A**. **Fig 3B** illustrates the inter-scanner agreement between MRF T_1_ relaxation times (bias 602.1 ms, 95% LOA 72.0 ms to 1132.0 ms) with only a single value being outside the 95% LOA. The outcomes of the Bland-Altman analysis for each individual tissue are presented in **Fig 4**, with acceptable agreement noted for all tissues included in the analysis. **S2** and **S3 Figs** depict the outcomes of a similar analysis for MRF T_2_ relaxometry.

**Fig 5:**
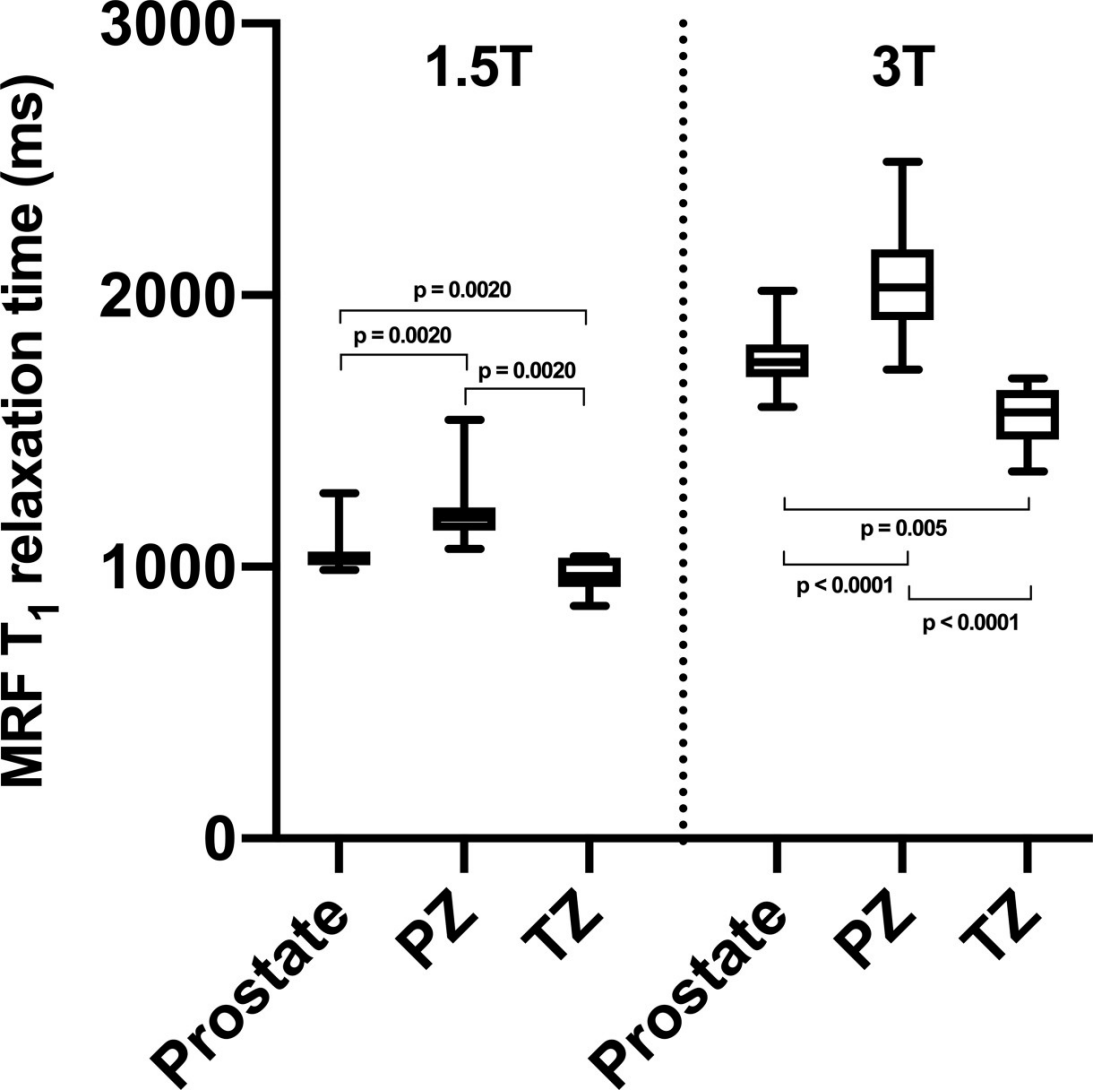
**Box-and-whiskers plots representing MRF T**_**1**_ **relaxation times obtained from the whole prostate, peripheral zone (PZ) and transition zone (TZ) at 1.5T and 3T (left and right sides of the graph, respectively).** Top and bottom of boxes represent 25^th^ and 75^th^ percentiles of data, respectively; line in boxes represents the median value and bars represent minimum and maximum values. MRF = magnetic resonance fingerprinting.

At both 1.5T and 3T, MRF T_1_ relaxometry enabled confident differentiation between the whole prostate, PZ and TZ (**Fig 5**). It is of note that the difference between MRF T_1_ relaxation times derived from PZ and TZ was slightly more prominent at 3T (24%) compared to 1.5T (19%). **S3 Fig** demonstrates the outcomes of a similar comparison for MRF T_2_ relaxometry.

## Discussion

This prospective proof-of-concept study investigated the cross-system reproducibility of SSFP MRF T_1_ of the prostate in healthy volunteers who were scanned using 1.5T and 3T systems with the same sequence parameters and reconstruction methods. The study was preceded by a phantom experiment that confirmed high reliability of MRF T_1_ mapping used when validated against IR-FSE imaging. The healthy volunteers study revealed acceptable inter-scanner agreement and excellent reproducibility of MRF T_1_ relaxometry in all tissues. At 3T, the magnitude of difference between MRF T_1_ obtained from the peripheral and transition zones of the prostate was slightly higher than at 1.5T, being, however, within the limits of statistical significance at both field strengths. These results, therefore, support further validation of prostate MRF at 1.5T as part of a standard-of-care mpMRI protocol.

To our knowledge, this is the first study to report MRF of the prostate performed at 1.5T, which is still commonly used for routine prostate imaging [26]. The observed high cross-system reproducibility of prostate MRF T_1_ is similar to that reported in the brain by Buonincontri *et al*., suggesting the overall robustness of MRF to field-dependent variation of T_1_ [30]. Our 3T MRF T_1_ values obtained from the healthy PZ and TZ are similar to those obtained in a patient population by other authors (2247.0 ms ± 450 ms for PZ [22] and 1800 ms ± 150 ms for TZ [20]) with subtle differences being expected due to the known age-related changes in histological and imaging features of the prostate [36, 37]. The observed ability of MRF to differentiate PZ and TZ in healthy volunteers at both 1.5T and 3T highlights MRF’s sensitivity to the underlying tissue organisation, which may be even more prominent in the older patients, in whom even visual differentiation of the prostatic zones is much less challenging [36, 38].

These preliminary results pave the way for future studies investigating the diagnostic performance of prostate MRF at 1.5T. It is known that prostate mpMRI at 1.5T is able to produce T_2_-weighted images of comparable diagnostic quality, however, the key argument in favour of 3T imaging is its ability to achieve higher signal-to-noise and contrast-to-noise ratios for diffusion-weighted imaging (DWI), which is the key sequence for PZ assessment, enabling DWI at higher b-values [39–42]. If 1.5T MRF retains its added value to ADC mapping previously demonstrated at 3T, it can potentially compensate for the inherent inferiority of DWI at 1.5T [20–22]. Furthermore, MRF’s inherent robustness to motion may help further establish its role as an auxiliary sequence to DWI, which is often degraded by motion artefact or rectal loading [43–46]. Finally, the added value of quantitative MRI can be harnessed clinically in the active surveillance setting where time-series change of tumour-derived relaxometry can serve as a more objective means of tracking the disease progression [47].

Our study has some limitations. The healthy volunteers study population was a small cohort of healthy volunteers of a younger age than typical patients with PCa. Although this may have introduced age-related heterogeneity, the reported high cross-system reproducibility of MRF is further complemented by its ability to differentiate normal PZ and TZ, which is considered less straightforward in younger adults [48]. Furthermore, the inclusion of older adults would limit our ability to sample healthy prostatic tissue due to high prevalence of benign conditions such as benign prostatic hyperplasia and prostatitis, or even undiagnosed PCa in otherwise healthy older men [49, 50]. To achieve comparable image quality, MRF at 1.5T was acquired with higher slice thickness and gap than at 3T, however, this mirrors standard clinical practice [51]. Having shown broadly similar results, MRF T_2_ mapping was unreliable in this study, and stronger consideration will be given in future studies to methods increasing its signal-to-noise ratio and improving the pattern matching algorithms.

In conclusion, the excellent reproducibility and high cross-system agreement of MRF T_1_ relaxometry of the healthy prostate observed in this preliminary study support the technique’s prospective clinical validation on 1.5T MRI systems. The reported findings could be used to justify further investigations for incorporating MRF into clinical prostate MRI protocols, larger population studies, and multi-site/multi-vendor investigations. If this method can be translated into clinical practice and improve both the speed and diagnostic value of imaging, it would add significant value given the current unprecedented demand on imaging services.

## Supporting information

S1 Table Summary MRF T_2_ relaxation times and coefficients of variation obtained at 1.5T and 3T from the whole prostate, peripheral zone, transition zone, internal obturator muscle and fat from the ischioanal fossa. SD = standard deviation.
(DOCX)

S1 Fig Linear regression (a) and Bland-Altman (b) plots comparing in vivo MRF T2 values obtained from all tissues combined at 1.5T and 3T systems. On Fig (b), dotted lines represent upper and lower 95% limits of agreement and bold lines represent the mean biases with appropriate captions included. MRF = magnetic resonance fingerprinting, SD = standard deviation.
(TIF)

S2 Fig Bland-Altman plots comparing in vivo MRF T2 values obtained from the whole prostate (a), peripheral zone (b), transition zone (c), internal obturator muscle (d) and fat in the ischioanal fossa (e) at 1.5T and 3T systems. Dotted lines represent upper and lower 95% limits of agreement and bold lines represent the mean biases with appropriate captions included. MRF = magnetic resonance fingerprinting, SD = standard deviation.
(TIF)

S3 Fig Box-and-whiskers plots representing MRF T2 relaxation times obtained from the whole prostate, peripheral zone (PZ) and transition zone (TZ) at 1.5T and 3T (left and right sides of the graph, respectively). Top and bottom of boxes represent 25th and 75th percentiles of data, respectively; line in boxes represents the median value and bars represent minimum and maximum values. MRF = magnetic resonance fingerprinting.
(TIF)
