# Supplementary material for: Reproducibility of magnetic resonance fingerprinting-based T1 mapping of the healthy prostate at 1.5 and 3.0 T: A proof-of-concept study
Source: PLoS One. 2021 Jan 29;16(1):e0245970. doi: 10.1371/journal.pone.0245970 (PMC7846281; doi:10.1371/journal.pone.0245970)
Supplement: S1 Table — SD = standard deviation. (DOCX) [file pone.0245970.s001.docx]

|  | Mean ± SD | | | Coefficient of variation (%) | |
| --- | --- | --- | --- | --- | --- |
| Tissue | **1.5T** | **3T** | ***P*** | **1.5T** | **3T** |
| Prostate | 282.5 ± 127.9 | 531.4 ± 187.6 | < 0.0001 | 45.26 | 35.30 |
| Peripheral zone | 329.0 ± 145.7 | 598.2 ± 218.5 | < 0.0001 | 44.28 | 36.52 |
| Transition zone | 211.8 ± 149.0 | 538.5 ± 221.1 | 0.0012 | 70.35 | 41.05 |
| Muscle | 160.2 ± 70.1 | 426.0 ± 180.5 | 0.0020 | 43.74 | 42.37 |
| Fat | 148.1 ± 7.5 | 176.2 ± 20.0 | 0.0017 | 5.05 | 11.37 |
