# Supplementary figures and images for: Reproducibility of magnetic resonance fingerprinting-based T1 mapping of the healthy prostate at 1.5 and 3.0 T: A proof-of-concept study
Source: PLoS One. 2021 Jan 29;16(1):e0245970. doi: 10.1371/journal.pone.0245970 (PMC7846281; doi:10.1371/journal.pone.0245970)

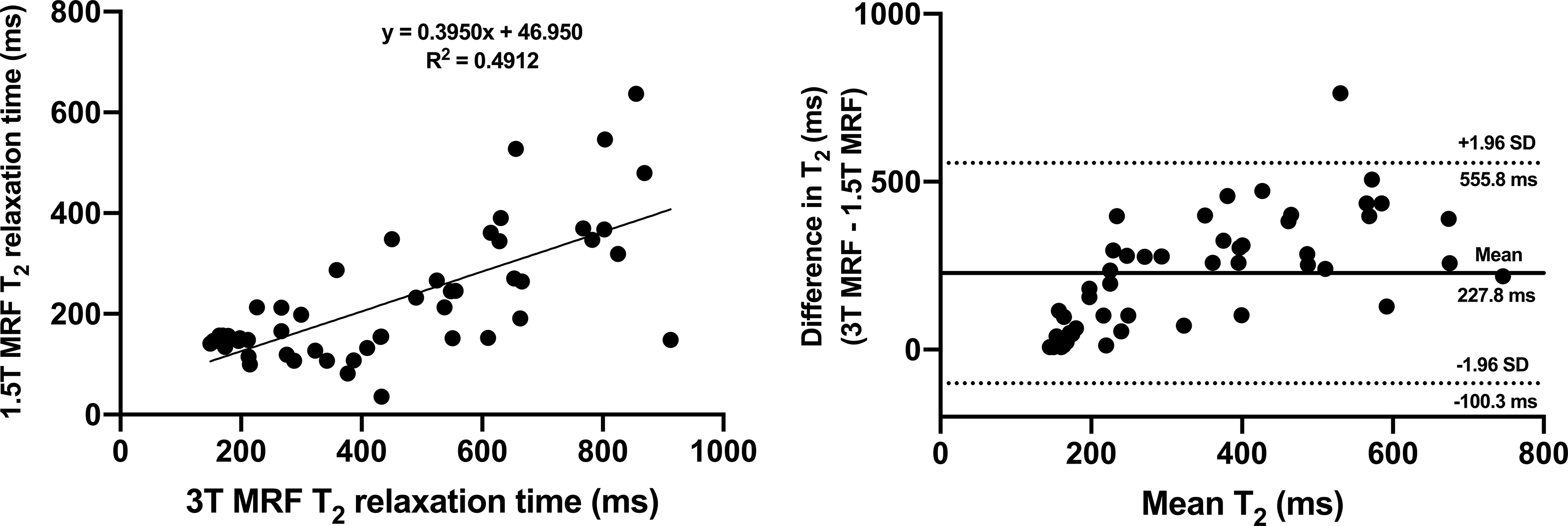

Supplement: S1 Fig — Linear regression (a) and Bland-Altman (b) plots comparing in vivo MRF T2 values obtained from all tissues combined at 1.5T and 3T systems. On Fig (b), dotted lines represent upper and lower 95% limits of agreement and bold lines represent the mean biases with appropriate captions included. MRF = magnetic resonance fingerprinting, SD = standard deviation. (TIF) [file pone.0245970.s002.tif]

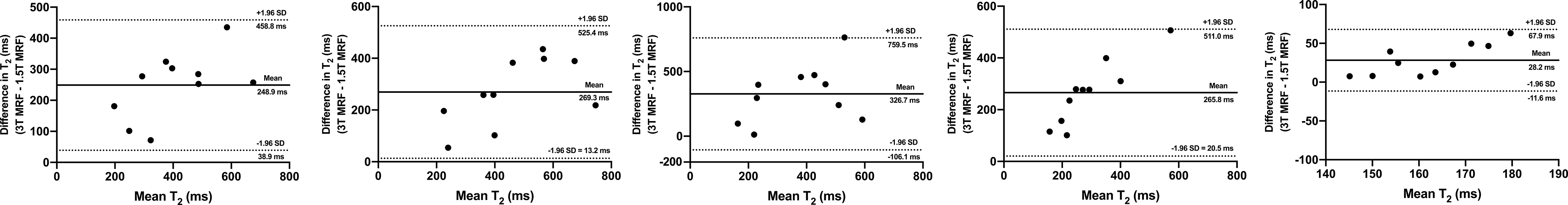

Supplement: S2 Fig — Bland-Altman plots comparing in vivo MRF T2 values obtained from the whole prostate (a), peripheral zone (b), transition zone (c), internal obturator muscle (d) and fat in the ischioanal fossa (e) at 1.5T and 3T systems. Dotted lines represent upper and lower 95% limits of agreement and bold lines represent the mean biases with appropriate captions included. MRF = magnetic resonance fingerprinting, SD = standard deviation. (TIF) [file pone.0245970.s003.tif]

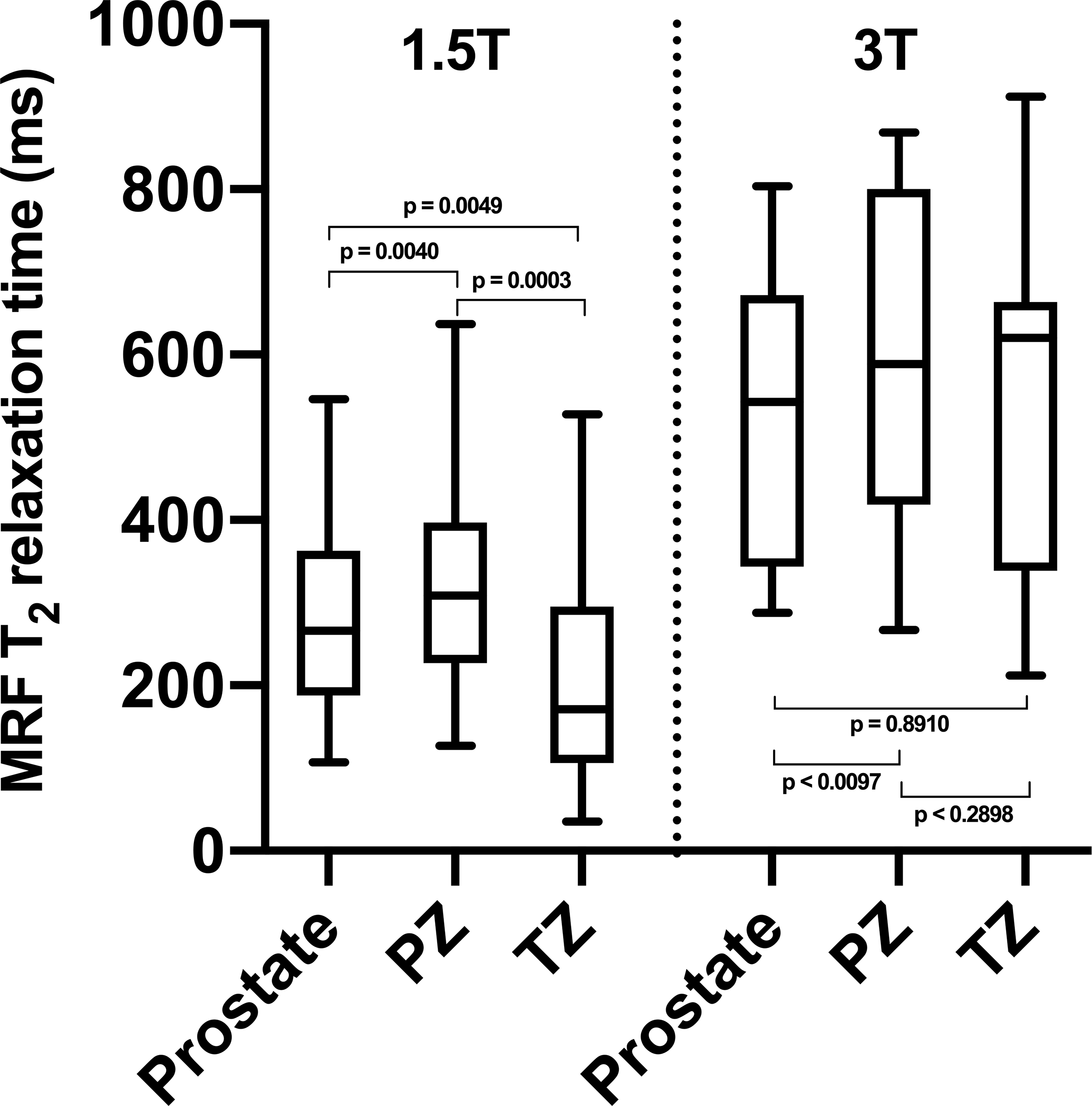

Supplement: S3 Fig — Top and bottom of boxes represent 25th and 75th percentiles of data, respectively; line in boxes represents the median value and bars represent minimum and maximum values. MRF = magnetic resonance fingerprinting. (TIF) [file pone.0245970.s004.tif]
